# A Rapid and Sensitive LC–MS/MS Method for Quantification of NDSRI in Melatonin Drug Product via Controlled In Situ Formation

**DOI:** 10.1002/bmc.70591

**Published:** 2026-08-14

**Authors:** Jaya Yalamanchili, Ganja Himavathi, Salakolusu Suresh

**Affiliations:** ^1^ Department of Chemistry, GITAM School of Science GITAM Deemed to be University Visakhapatnam Andhra Pradesh India; ^2^ Analytical Discovery Chemistry Aragen Life Sciences Pvt. Ltd. Hyderabad India

**Keywords:** liquid chromatography‐tandem mass spectrometry (LC–MS/MS), melatonin, multiple reaction monitoring (MRM), nitrosoamine drug substance–related impurities (NDSRIs)

## Abstract

Nitrosamine drug substance–related impurities (NDSRIs) pose a significant safety risk due to their mutagenic and carcinogenic potential, prompting strict regulatory oversight under ICH M7. The propensity for in situ formation of NDSRIs during analytical procedures, particularly in active pharmaceutical ingredients containing reactive amines, emphasizes the need for highly accurate and scrupulously validated methodologies. Melatonin, containing a vulnerable amine, was chosen as a model drug product. A liquid chromatography–tandem mass spectrometry (LC–MS/MS) method was developed and validated for quantifying N‐Nitroso melatonin. To prevent in situ formation, a scavenger was incorporated during sample preparation. Validation followed regulatory standards, assessing specificity, linearity, accuracy, precision, sensitivity, and robustness. The method showed excellent linearity (1.8–22.5 ng/mL, *R* ^2^ = 0.9983), sensitivity with LOD of 0.9 ng/mL and LOQ of 1.8 ng/mL, and accuracy with recoveries between 85.3% and 95.9%. Precision was confirmed with %RSD below 2%, whereas robustness testing demonstrated consistent performance under varied conditions. Crucially, the scavenger eliminated false positives by suppressing in situ nitrosamine formation, ensuring data integrity. This validated approach strengthens analytical accuracy and provides a transferable framework for monitoring NDSRIs in other drug products containing reactive amines, supporting regulatory compliance and safeguarding patient health.

## 1 Introduction

Melatonin is a ubiquitous molecule synthesized naturally and majorly secreted by the pineal gland in the human body. The skin, bone marrow, gut, ovaries, and prostate are just a few of the peripheral sites where this neuroendocrine substance is produced and released. Under typical environmental circumstances, the production of melatonin increases in the evening, causing sleeplessness during the day and initiating a state of sleep at night. Depending on the light schedule, light may either suppress or synchronize melatonin production. The main physiological function of the hormone melatonin is to transmit information about the daily cycle of light and darkness to the body's physiology. In response to the light/dark cycle, the suprachiasmatic nuclei produce the body's own rhythm of secretion. The daily melatonin secretion, which serves as a strong biochemical indication of night, may be utilized to regulate circadian rhythms. Although melatonin interacts through MT1 and MT2, which are G‐protein coupled melatonin receptors, it has also been shown that melatonin is a potent antioxidant and has a significant impact on the cell cycle (Al‐Ansari et al. 2024; Pohanka 2022; Sweis 2005; Sanchez‐Barcelo et al. 2010; Jørgensen et al. 2022; Savage et al. 2024; Hattori et al. 1995; Reiter et al. 2001; Claustrat et al. 2005; Ahmad et al. 2023). The American Academy of Family Physicians (AAFP) considers melatonin to be the first‐line pharmacological treatment for insomnia, highlighting its vital function in addressing sleep‐related anxieties. Additionally, melatonin is used in the treatment of jet lag, neurodegenerative diseases, posttraumatic brain injury, and migraine prevention. Despite the fact that the US Food and Drug Administration (FDA) has not formally approved melatonin for any use in the nation, melatonin, which is sold as a synthetic nutritional supplement, mirrors the regulatory actions of the body's own melatonin. Despite the fact that the roles of melatonin in people are mostly based on correlational studies, there is some data suggesting that it stabilizes and reinforces the connection between circadian rhythms, particularly the primary temperature and sleep–wake cycles. The secretion of melatonin is essential for the daily management of various physiological processes, including glucose regulation, hemostasis, immunological, and antioxidant defenses. Additionally, melatonin has anti‐inflammatory and anti‐excitatory actions in addition to its antioxidant properties. Melatonin is shown to have a wide range of additional activities, including transplantation, puberty timing, reproduction, immunological regulation, hypnotic, oncostatic, and mood problems. A deficiency in melatonin production is linked to the beginning of a number of illnesses, including breast cancer and neurodegenerative diseases. In diseases related to pain, melatonin has a silent but significant role as a possible pain reliever.

The detection of nitroso dimethylamine (NDMA) in one of the drug products has led to severe concerns for presence of nitrosamine impurities in pharmaceutical products in 2018. This discovery laid foundation to impose high quality standards for drug substance and drug products through stringent acceptable intake levels to avoid product recalls (U.S. Food and Drug Administration 2022; European Medicines Agency 2021; European Medicines Agency 2026). Even though it started with simple nitrosamines in the beginning, but later transformed to complex nitrosamines with high mutagenic and carcinogenic effect that include nitrosamine drug substance–related impurities (NDSRIs). NDSRIs are structurally similar to the corresponding drug substance and these are formed when nitrosating agents interact with the active pharmaceutical ingredients (APIs) containing amine groups or their byproducts under specific environmental conditions. However, for most of NDSRI's, it became difficult to specify suitable acceptable intake limits due to lack of respective toxicity data. The consequence of this made several regulatory agencies to propose AI limit of 18 ng/day for all the NDSRIs whose carcinogenic data is not available by considering a worst‐case scenario. However, meeting these stringent limits poses several challenges even after using state‐of‐the‐art technology. In the nitrosamine evolution process, a new approach is proposed for better classification of NDSRIs into suitable AI limits ranging from 18 to 1500 ng/day. Based on this approach, i.e., carcinogenic potency categorization approach (CPCA), that classifies nitrosamine based on their structural relationship to carcinogenic potency, several NDSRIs were categorized (Health Canada 2024; U.S. Food and Drug Administration 2023a; International Council for Harmonisation [ICH] 2018; Cioc et al. 2023; Ashworth et al. 2020). In order to meet the AI limits and corresponding lower limit of quantification limits, a robust and sensitive method is required for accurate quantification of NDSRI in drug product, which is achieved preferably using state of the art hyphenated techniques.

The close resemblance between NDSRI and API structures poses several challenges in quantification methods in terms of separation and also owing to high API concentrations to meet the sensitivity requirements. In addition, the presence of a more vulnerable amine group in API and nitrite sources from environmental conditions will favor the in situ NDSRI formation. In situ NDSRI formation means, even though the drug product itself does not contain any NDSRI content, during the sample preparation step in quantification methods there are chances for NDSRI formation owing to the presence of nitrite in trace levels from the solvents or chemicals used for sample preparation (Wichitnithad et al. 2023; Baumann and Näf 2024; Golob et al. 2023; Ashworth et al. 2020; International Council for Harmonisation [ICH] 2023).

**FIGURE 1:**
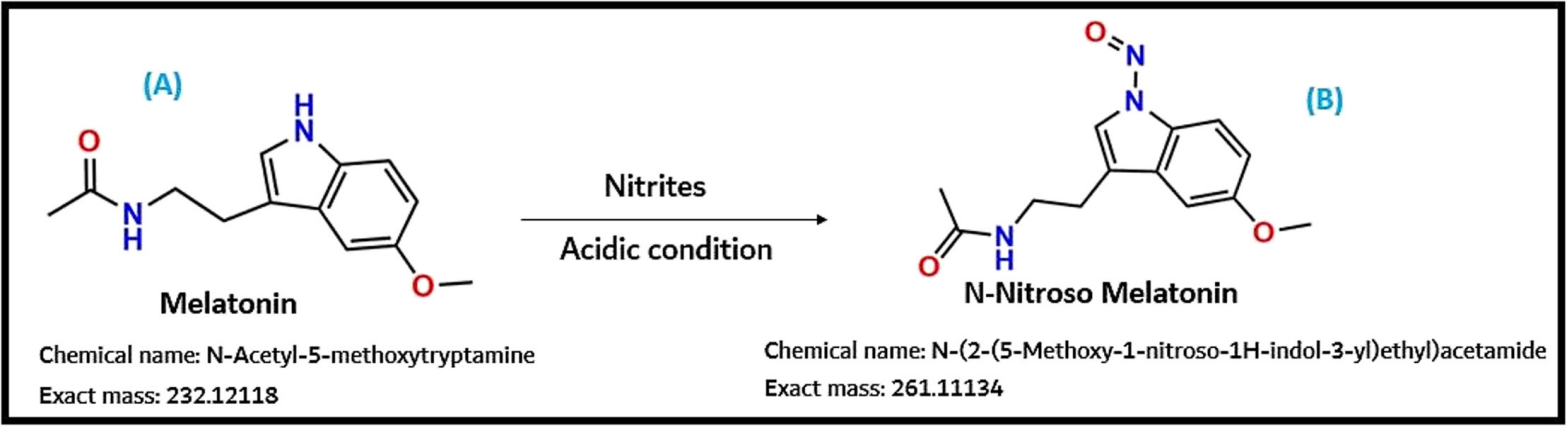
Formation of N‐Nitroso melatonin from melatonin. (A) Melatonin. (B) N‐Nitroso melatonin.

This will seriously affect the accuracy of the data with false positive results and lead to unsuccessful and time‐consuming mitigation strategies. The aim of this research is to develop a sensitive and accurate LC–MS/MS method for quantification of N‐Nitroso melatonin (NMLT) in melatonin drug product (Figure 1). The developed method should be capable of effectively controlling the in situ NDSRI formation. This analytical method should be proved to be appropriate for its intended purpose through a set of experiments by considering the validation requirements as per the ICHQ2 (R1) guidelines (International Council for Harmonisation [ICH] 2005; Joint Committee for Guides in Metrology [JCGM] 2008; Cécile et al. 2023). This method will help to enhance the safety and quality of pharmaceutical products by meeting the regulatory requirements especially for reporting NDSRIs in accurate levels.

## 2 Experimental Section

### 2.1 Chemicals and Reagents

Solvents including water, methanol, and acetonitrile used for sample preparation and analysis were of LC–MS grade and purchased from Biosolve. Chemicals including ammonium formate and 3,4‐diamino toluene were purchased from Sigma‐Aldrich chemicals. Melatonin tablets were purchased from a local pharmaceutical drug store. Reference standard of NMLT was obtained from SynZeal manufacturer. PVDF syringe filters from Whatman were used for sample clarification.

### 2.2 HPLC Conditions

For chromatographic separation, an HPLC system made by Shimadzu, which comprises a pump, autosampler, column oven, and UV detector, was used. XBridge BEH Shield RP18 Column (3.0 × 150 mm, 2.5 μm) maintained at 40°C was used as the stationary phase for efficient separation. An injection volume of 10 μL was used for analysis, and the autosampler vial temperature was maintained at 10°C. The mobile phase consists of 10 mM ammonium formate in water (Eluent‐A) and acetonitrile and methanol at an 80:20 ratio (Eluent‐B) with a flow rate of 0.6 mL/min. The gradient program is 10% B (0–3.0 min); 10%–80% B (3.5–9.0 min); 80%–95% B (9.5–14.0 min); 95% B (14.0–18.0 min) and equilibration at 10% B (18.5–20 min).

### 2.3 Mass Spectrometer Conditions

The LCMS instrument from the Sciex mass spectrometer model QTrap 4500 is used for LC–MS/MS analysis, which is interfaced with the HPLC system. Among electrospray ionization (ESI) and atmospheric pressure chemical ionization (APCI) modes, Turbo spray ESI mode was used with positive ion polarity. Scan type of multiple reaction monitoring (MRM) was used with transition monitored at *m*/*z* 262.2 → 173.1 for NMLT. Collision energy of 10 was applied for NMLT for stable and high intense product ions of 173.1. The declustering potential of 30 and source temperature of 500°C were optimized. The gas flow of 40 and 50 were selected for GS1 and GS2, respectively. The initial eluent was diverted to waste using a switching valve up to 13 min run time for minimizing the source contamination with melatonin ions. Data acquisition and processing were performed using Analyst software.

### 2.4 Preparation of Standard Solution

#### 2.4.1 Stock Solutions

Standard stock solution of NMLT was prepared at 0.9 mg/mL in methanol.

#### 2.4.2 Intermediate Stock Solution

An intermediate stock solution was prepared using the stock solution of NMLT at a concentration of 0.9 μg/mL in methanol. Transferred 100 μL of the standard stock solution into a 100‐mL volumetric flask and diluted it up to the mark with methanol.

#### 2.4.3 Working Concentration Solution

Intermediate stock solution, then diluted with methanol to a working concentration of 90 ng/mL. Transferred 1000 μL of intermediate stock solution into 10 mL volumetric flask and diluted up to the mark with methanol.

#### 2.4.4 Standard Solution

A series of calibration standards were prepared from working concentration solution with concentrations of 1.8, 9.0, 13.5, 18.0, and 27.0 ng/mL. These concentrations would represent 0.18, 0.90, 1.35, 1.8, and 2.7 ppm, respectively, when calculated with respect to the sample concentration. In each preparation, 1 mL of 1 M 3,4‐diamino toluene solution was added before making up the solution to the final volume with a 90% methanol in water solution. The 100% standard, defined as the specification limit, was 18.0 ng/mL. Recovery experiments involved preparing NMLT at 1.8, 18.0, and 27.0 ng/mL in the drug product samples.

### 2.5 Sample Preparation

Melatonin tablets were crushed to fine powder using mortar and pestle and the powder was used for sample preparation. A sample equivalent to 50 mg (sample concentration: 10 mg/mL) of melatonin was transferred into a 15 mL centrifuge tube. Added 4 mL of 90% methanol in water and mixed the solution and 1 mL of 1 M 3,4‐diamino toluene solution. Vortexed the solution for 3 min using a mechanical vortex. The solution was centrifuged at 3000 rpm for 5 min and the upper clear layer was filtered through a 0.22 μm PVDF syringe filter (Whatman), discarding the initial 3 mL. A 1 mL aliquot of the filtered solution was then carefully transferred to an autosampler vial and used for LC–MS/MS analysis. A blank was prepared similar to the above procedure, except for the addition of the sample.

#### 2.5.1 Method Validation

The analytical method for NMLT was validated according to ICH Q2 (R1) guidelines to show that the method is fit for its intended use, with validation parameters including specificity, linearity, limit of detection (LOD) and limit of quantitation (LOQ), precision, accuracy, and robustness.

Specificity of method was assessed using blank, individual standard solutions, and drug product to verify the absence of interference at retention time for the analyte of interest. LOD was determined based on signal‐to‐noise ratio (S/N) of ≥ 3 and LOQ was determined based on S/N of ≥ 10, respectively. Linearity was evaluated by performing a single analysis at each concentration level across the range of 0.18–27 ng/mL (corresponding to 10%–150% of the limit). Residual plot analysis and *R* ^2^ values exceeding 0.998 confirmed the linearity of the method. Accuracy and precision were evaluated by analyzing the samples spiked with NMLT. The accuracy was conducted using triplicate analyses at three concentrations (1.8, 18, and 27 ng/mL, i.e., 0.18, 1.8, and 2.7 ppm with respect to sample concentration) within the calibration range. For each concentration level, the mean assay values, percent recoveries, and relative standard deviations (%RSD) for recoveries were calculated. As part of method robustness assessment, the %RSD of peak response and retention time for NMLT was measured after modifying the eluent flow rate and column temperature by ±10%.

### 2.6 Specification Limit

NMLT does not have established toxicity data and is not currently included in any of the CPCA schemes. Due to absence of compound specific carcinogenicity data and continuous update of guidelines towards more stringent AI limits than the previously published limits, the default AI limit of 18 ng/day as per EU guideline which constitute the lowest limit for NDSRIs was considered in specifying the limit (Dobo et al. 2022; Kalgutkar et al. 2023; U.S. Food and Drug Administration 2023b, updated 2025). This lower AI limit will further greatly serve the purpose of in situ NDSRI formation and corresponding control strategies which impact more at lower‐level method requirements. To derive the permissible specification limit for NMLT in melatonin drug products, the AI value (ng/day) was normalized to the maximum daily dose of melatonin (mg/day). Thus, considering the maximum daily dose of melatonin as 10 mg/day, the specification limit for NMLT is 1.8 ng/mg or ppm (Shenoy et al. 2024). This threshold was subsequently adopted as the working limit during method development and validation studies to ensure compliance with regulatory expectations and to provide a scientifically justified basis for analytical control of NMLT in melatonin drug product.

## 3 Results and Discussion

### 3.1 Method Development

**FIGURE 2:**
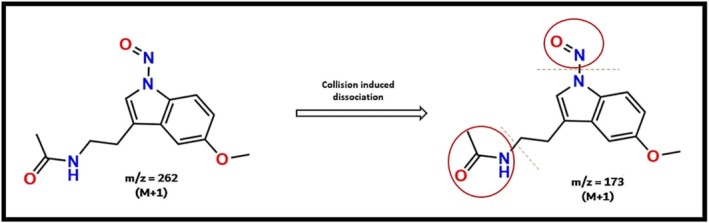
Dissociation of N‐Nitroso melatonin under collision induced dissociation.

**FIGURE 3:**
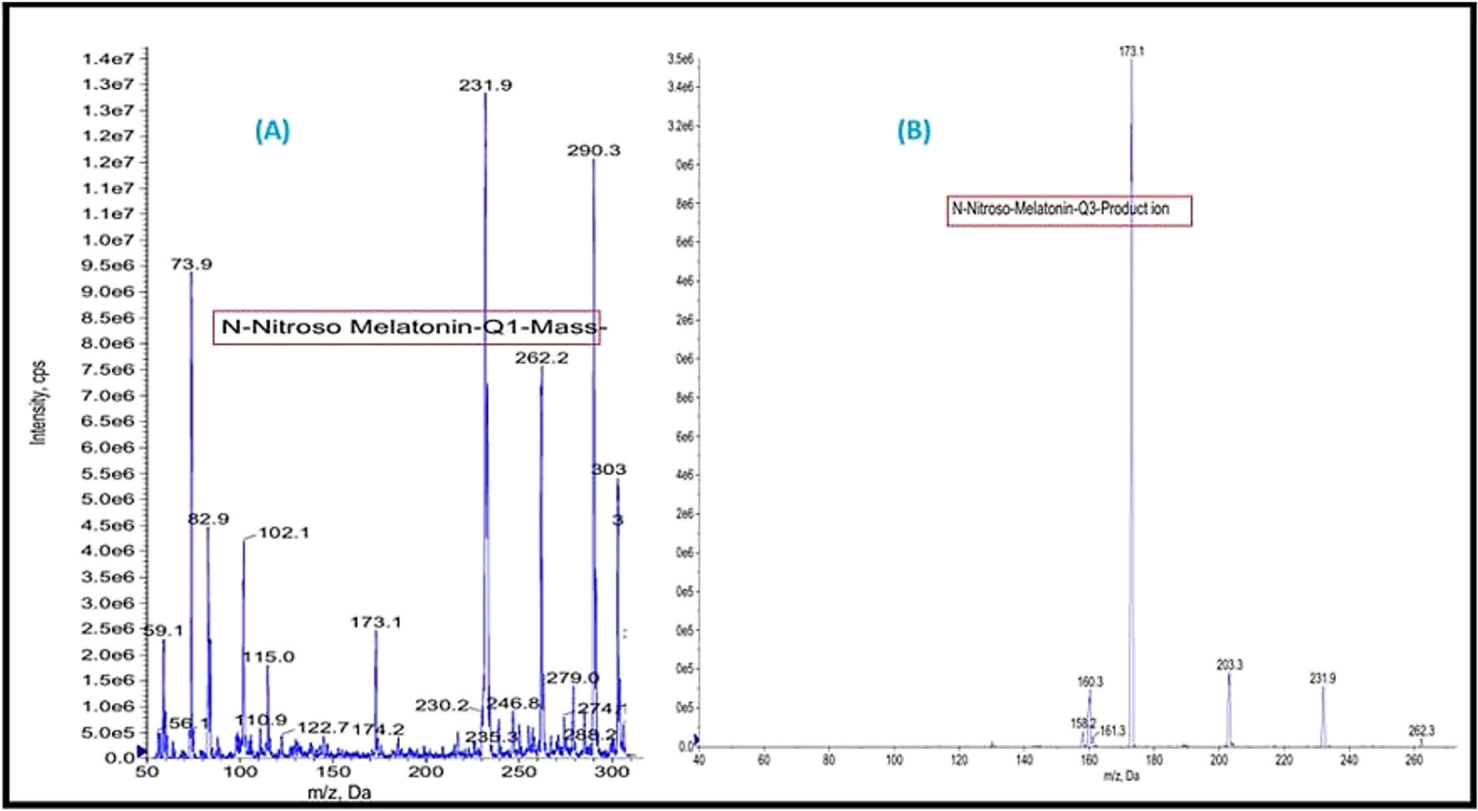
Mass spectral data. (A) N‐Nitroso melatonin Q1 scan. (B) N‐Nitroso melatonin Q3 product scan.

MS parameters were optimized for NMLT by initially infusing NMLT solution at 5 μg/mL concentration level through tuning window. Identified the molecular ion of NMLT as 262.2 m/z in ESI positive mode. No significant mass was observed in negative ionization mode compared with positive ionization mode. Hence, ESI positive mode was selected for quantification. Evaluated the dissociation by applying the collision‐induced dissociation to get the product ions. The collision energy was applied from 8 to 30 and identified that at collision energy of 10 eV applied for NMLT a stable and high intense product ion of 173.1 was observed. Further, optimized the source parameters like declustering potential, source temperature, and cone voltage. The declustering potential and source temperature were optimized and observed that the product ion is exhibiting high signal intensity and reproducibility at declustering potential and source temperature of 30 and source temperature of 500°C, respectively (Figure 2). Based on these findings, the transition m/z 262.2 → 173.1 was selected for MRM mode, thereby ensuring enhanced sensitivity and specificity for quantitative analysis of NMLT (Figure 3). Method development using LC–MS was initiated using a C18 stationary phase with an initial mobile phase composition of 0.1% formic acid in water and methanol. Under these chromatographic conditions, melatonin was eluted prior to NMLT. To achieve a sufficient retention time separation between the two analytes, essential for accurate and distinct quantification, the mobile phase composition and gradient program were methodically optimized. Alternative stationary phases, including phenyl and C8 columns, were evaluated; however, these did not provide any significant improvement in retention time separation relative to the C18 column. Based on these findings and considering practical factors such as widespread availability and reproducibility, the RP18 column was confirmed as the most suitable choice for the separation of melatonin and NMLT. To prevent mass spectrometer saturation at source due to the high sample concentration, the flow was diverted to waste during the first 13 min and after 19 min of the chromatographic run. Based on the flow rate of 0.6 mL/min, the gas flow of 40 and 50 were selected for GS1 and GS2, respectively. Once the required separation and sensitivity were achieved, further the method accuracy studies were initiated as part of method development. During the course of sample analysis, a critical abnormality was encountered that posed a significant challenge to method development. Specifically, inconsistent peak areas were observed across different sample preparations, despite adherence to identical analytical conditions and sample preparation steps. Notably, this irregularity was not observed in the case of standard injections, even when evaluated at varying concentration levels, thereby ruling out instrumental or calibration‐related issues. The inconsistency in sample responses subsequently revealed as aberrant recovery values during spike recovery experiments, highlighting a matrix‐related effect that is hindering the establishment of a robust and reproducible quantification method.

A systematic evaluation was initiated to identify the possible contributing factors for the inconsistent areas in analysis. Among the potential causes, one possibility was the in situ formation of NDSRIs during sample preparation process. To verify this possibility, a controlled experiment was performed by deliberately adding known quantity of sodium nitrite solution during sample preparation step (Azeez et al. 2018; Sharma et al. 2023; Patel et al. 2025; Carlos et al. 2025; Mukherjee et al. 2025; Gopireddy et al. 2024). This will enhance the nitrite availability in solution to react with API in sample. As expected, the API in the sample reacted with the nitrite and formed the NDSRI, which is evident from the peak area in this sample injection. In as such sample preparation, there is no NMLT peak was observed, whereas after addition of nitrite conditions, a peak was observed in MRM with content of 50 ppm. This confirmed that even trace levels of nitrite present in the diluent could induce in situ NDSRI formation, thereby leading to inconsistent peak areas and compromised quantification.

**FIGURE 4:**
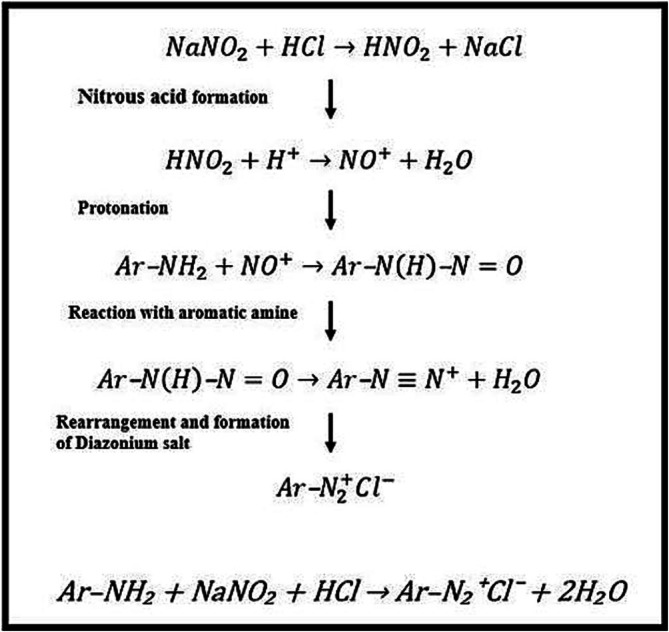
Reaction mechanism of aromatic amine with nitrites.

**FIGURE 5:**
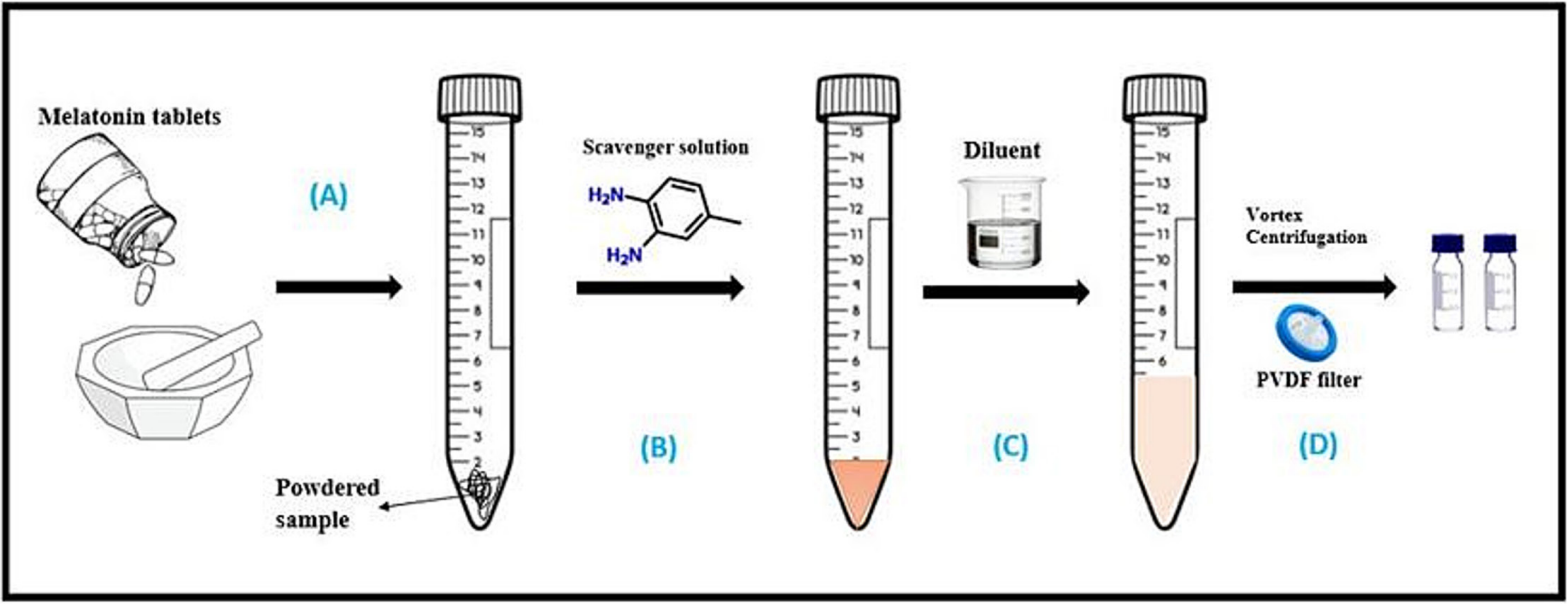
Sample preparation procedure. (A) Grinding of samples to fine powder. (B) Addition of 3,4‐diamino toluene solution. (C) Final volume makes up. (D) Filtration.

Following identification of the root cause, various reagents containing strong amine functional groups were evaluated for their ability to interact with trace nitrites in the diluent and thereby prevent their reaction with the API. The chemicals include trinitrotoluene, 2,4,6‐trinitrophenol, 3,4‐diamino toluene, and 2,6‐dinitroanisole. Among these, all chemicals come under toxic and explosive categories except 3,4‐diamino toluene. Among these, 3,4‐diamino toluene was selected as the most suitable scavenging agent based on its favorable physical and chemical properties. The introduction of 1 M 3,4‐diamino toluene solution into the sample preparation before addition of diluent to the sample matrix, resulted in consistent recoveries. The samples were grinded to powder; to this, 3,4‐diamino toluene solution was added, followed by the addition of diluent until reaching the required dilution. Subsequently vortexed sample, centrifuged, and filtered using PVDF filters (Figure 4). The reaction mechanism of aromatic amines with nitrites, in this case, 3,4‐diamino toluene reacts with traces of nitrite present in diluent and forms diazonium salts (Figure 5). Thus, the method was finalized with the optimized sample preparation procedure. Based on results of preliminary assessment for precision and accuracy, the method was finalized and subsequently subjected to comprehensive validation as per ICHQ2 (R1) guidelines.

### 3.2 Validation Studies

#### 3.2.1 Specificity

**FIGURE 6:**
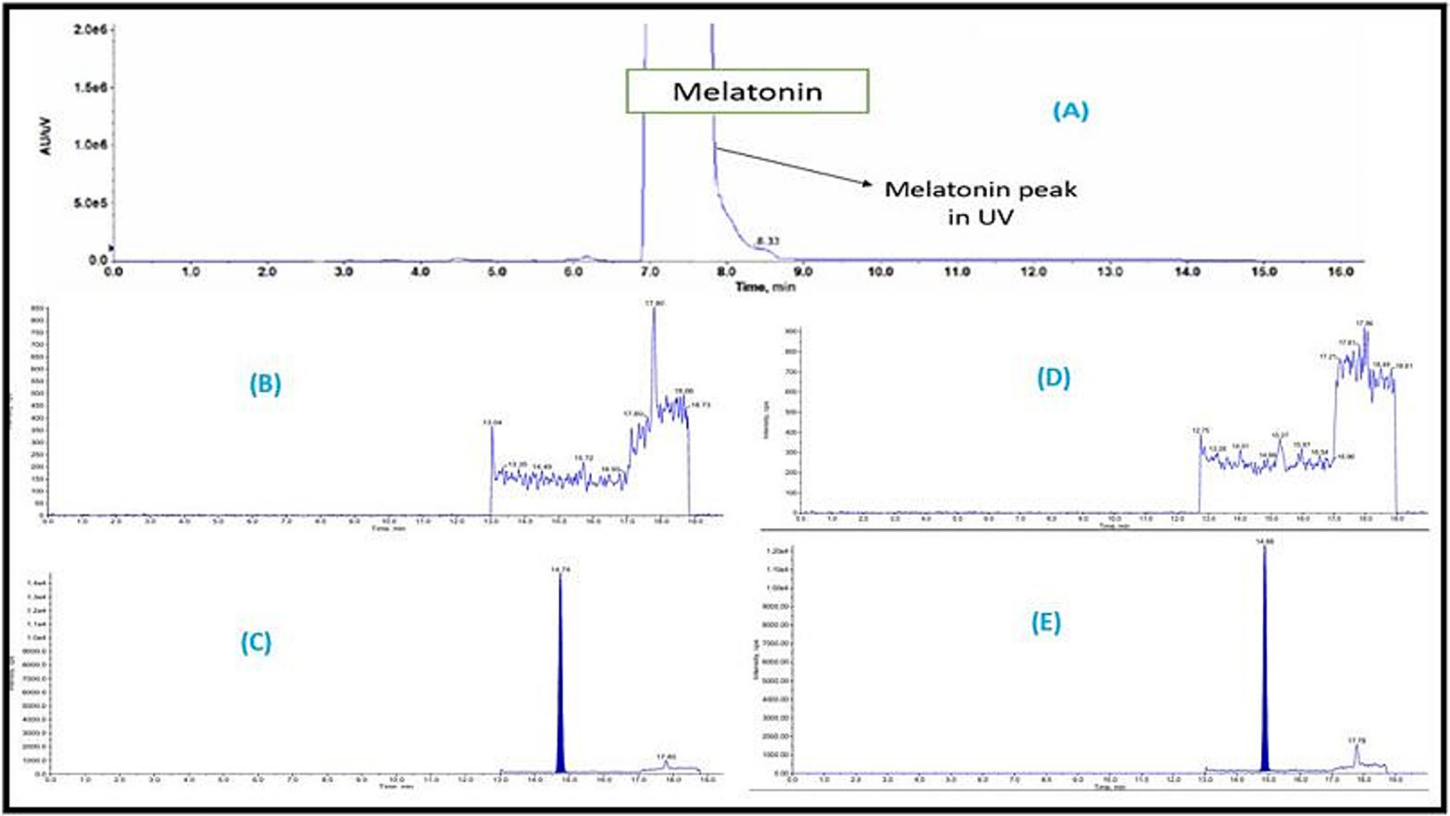
Specificity data for N‐Nitroso melatonin. (A) Melatonin in UV data. (B) MRM data in blank injection. (C) MRM data in sample injection. (D) MRM data in standard injection. (E) MRM data in sample spiked with N‐Nitroso melatonin injection.

Specificity of this method was established by comparing the MRM data of diluent with standard solution and drug product solution. Evaluated the data for presence of any interference in blank at the retention time of NMLT peak. It revealed that there are no interfering substances at the retention time of the analyte peak, thus confirming that the method is specific to NMLT (Figure 6).

#### 3.2.2 Linearity

Linearity of the analytical method was rigorously evaluated across the expected concentration range of NMLT. A linear calibration model was constructed using five standard solutions prepared at concentrations of 1.8, 9.0, 13.5, 18.0, and 27 ng/mL. This range was deliberately selected to include 10%–150% of the target specification, thereby ensuring that the calibration adequately represents both lower and higher levels relative to the sample concentration. The linear plot with concentration on the x‐axis and response on the y‐axis was considered for linearity assessment. The resulting regression analysis demonstrated excellent linearity, with a correlation coefficient (*R* ^2^) of 0.9983, indicating a near‐perfect fit of the data to the linear model. The regression equation (*y* = 531,548*x* − 33,372) further supports the robustness of the calibration. Collectively, these findings confirm that the method exhibits strong linearity and is suitable for accurate quantification of NMLT within the validated concentration range (Data S1).

#### 3.2.3 LOD and LOQ

**FIGURE 7:**
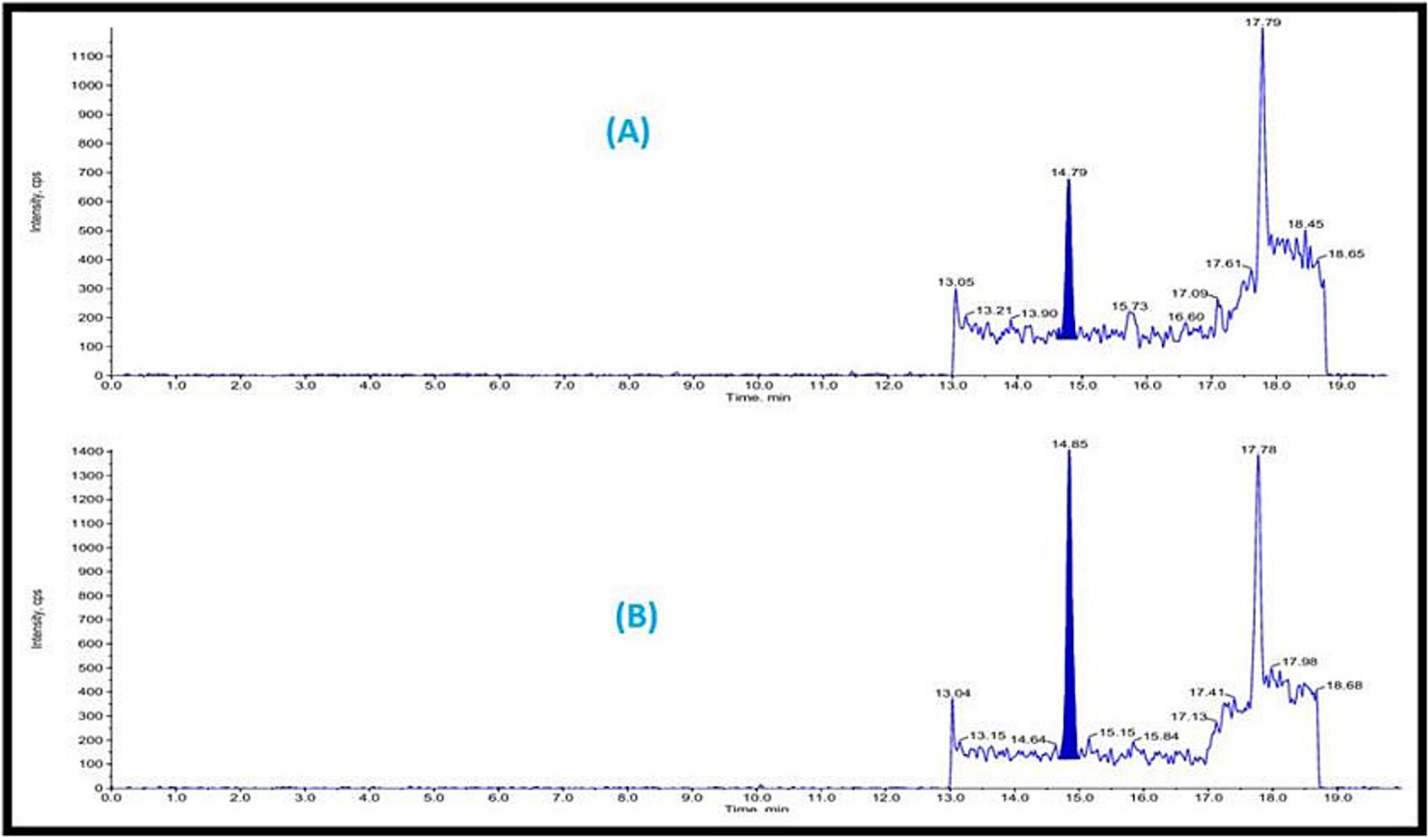
Sensitivity of N‐Nitroso melatonin. (A) MRM data of N‐Nitroso melatonin at LOD concentration level. (B) MRM data of N‐Nitroso melatonin at LOQ concentration level.

The sensitivity of the analytical method was established by determination of the LOD and LOQ using the S/N approach. This strategy was selected in alignment with regulatory expectations, ensuring that the method determines capability at the lowest achievable limits of measurement. Method optimization was focused on achieving at least an S/N of ≥ 3 for LOD and ≥ 10 for LOQ, consistent with internationally accepted validation criteria. The optimized conditions successfully met these thresholds, confirming an LOD of 0.9 ng/mL (0.09 ng/mg) and an LOQ of 1.8 ng/mL (0.18 ng/mg). These values confirm that the method possesses sufficient sensitivity to reliably detect and quantify trace levels of NMLT within the tested matrix. The attainment of such low limits emphasizes the robustness of the procedure and its suitability for regulatory submission, as it ensures accurate monitoring of analyte concentrations even at levels approaching the lower boundary of specification (Figure 7).

#### 3.2.4 Precision

Precision of the analytical method was evaluated through assessment of both repeatability (method precision) and intermediate precision parameters. Method precision was determined by preparing and analyzing six independent sample preparations of melatonin spiked with NMLT at 100% of the specification limit. The relative standard deviation (%RSD) of the measured content across these replicates was calculated and found to be 0.7. Thereby demonstrating excellent repeatability and minimal variability under identical conditions. Intermediate precision was further assessed by repeating the experiment on a subsequent day using the same instrument and freshly prepared samples. The overall % RSD for both days was calculated and found to be 1.8 for NMLT. The consistency of results across different days confirmed that the method is robust and reproducible, with negligible day‐to‐day variation. Collectively, these findings establish that the method possesses high precision, meeting the stringent requirements for quantitative determination of NMLT in the drug product matrix and preparations (Table 1; Supporting Information S1: Supporting data‐1).

**TABLE 1:** Validation results.

Validation parameter	Acceptance criteria	Result obtained	Conclusion
Specificity	No interference from matrix	Clear baseline	Complies
Precision (repeatability)	%RSD ≤ 20%	0.7%	Complies
Intermediate precision	%RSD ≤ 25%	1.8%	Complies
Linearity	*R* ^2^ ≥ 0.99	0.9983	Complies
Range	Between 10%–200% of test concentration	10%–150%	Complies
Limit of detection (LOD)	Signal‐to‐noise ≥ 3	0.09 ppm	Complies
Limit of quantitation (LOQ)	Signal‐to‐noise ≥ 10	0.18 ppm	Complies
Robustness	No significant change in results	Consistent	Complies
Accuracy	70%–130% recovery	90.7% at LOQ	Complies
		95.6% at 100% specification level	
		86.5% at 150% specification level	

#### 3.2.5 Accuracy

The accuracy parameter was expressed in terms of % recoveries and precision expressed as %RSD of the NMLT and these were evaluated in triplicate at 10% (LOQ), 100%, and 150% of the specification limit, using drug product. Recovery calculations were conducted without correction factors, as the sample matrix did not exhibit any endogenous peaks corresponding to NMLT, thereby eliminating potential bias. The assay yielded recovery values ranging from 85.3% to 95.9%, demonstrating acceptable accuracy across the tested range. Precision was confirmed by %RSD values below 2% at all levels, including LOQ, 100%, and 150% of the specification limit, indicating excellent reproducibility of the measurements. These findings establish that the method provides reliable, accurate, and precise quantification of NMLT in the drug product matrix, meeting the stringent requirements for analytical validation (Table 1; Supporting Information S1: Supporting data‐1).

#### 3.2.6 Robustness

To evaluate the robustness of the method, key chromatographic parameters were selected and applied slight but significant variations. These included adjusting the mobile phase flow rate by ±0.5 mL/min and modifying the column temperature by ±5°C. NMLT demonstrated a consistent peak area, with %recovery values remaining within acceptable limits. These results confirm that the method maintains a reliable performance despite minor fluctuations in operating conditions (Table 1).

#### 3.2.7 Matrix Effect

The matrix effect involves comparison of data with matrix and without matrix to understand the effect of matrix on the response of analyte. This matrix effect is studied by considering the slope ratio calculation with and without matrix (Nakka, Marisetti, and Manabolu Surya 2025; Nakka, Katari, et al. 2025). A slope ratio between 0.85 and 1.15 was interpreted as indicative of an insignificant matrix effect, suggesting that the matrix components exerted minimal influence on the analytical signal and overall quantification accuracy. Three levels that are common for both linearity and accuracy, i.e., 10% (LOQ), 100%, and 150% of the specification limit injections were considered, and the slope was calculated individually. The slope ratio of 0.87 indicates matrix effect is within the range of 0.85–1.15. These results indicates that there is no significant matrix effect on the NMLT peak, further confirming the accuracy of data.

## 4 Conclusions

NDSRIs are classified under the ICH M7 “Cohort of Concern” due to their high mutagenic and carcinogenic potential, necessitating stringent control at trace levels to safeguard patient health. A critical challenge in their quantification is the risk of in situ NDSRI formation during analytical procedures, particularly in APIs containing reactive and vulnerable amine functionalities. In this study, a highly sensitive LC MS/MS method was developed and validated for the quantification of N‐Nitroso melatonin in melatonin drug products. The method was strategically optimized by incorporating a scavenger during sample preparation to suppress in situ NDSRI formation, thereby ensuring accurate quantification and eliminating false positives. Validation parameters including specificity, precision, accuracy, linearity, and sensitivity (LOD and LOQ) demonstrated that the method is reliable, reproducible, and fit for routine application. The successful establishment of this approach not only enables accurate monitoring of NMLT but also provides a framework for mitigation and control strategies in pharmaceutical analysis. Importantly, the methodology exemplifies effective management of in situ NDSRI formation during analytical testing, thereby enhancing confidence in data integrity. Beyond its immediate application to melatonin, this validated LC–MS/MS approach demonstrates potential usefulness for other drug products containing reactive amines, with individual compound‐specific validation. The method provides a valuable reference framework to support regulatory compliance and enhance proactive safety management in controlling NDSRI formation. Overall, the study underscores the importance of robust analytical strategies in ensuring patient safety by enabling precise quantification of trace nitrosamine impurities and supporting the development of risk mitigation measures in pharmaceutical quality control.

## Funding

The authors have nothing to report.

## Ethics Statement

The authors have nothing to report.

## Conflicts of Interest

The authors declare no conflicts of interest.

## Supporting information


**Data S1:** Supporting information.

## Data Availability

All data generated or analyzed during this study are included in this published article.
