# Supplementary material for: A Rapid and Sensitive LC–MS/MS Method for Quantification of NDSRI in Melatonin Drug Product via Controlled In Situ Formation
Source: Biomed Chromatogr. 2026 Aug 14;40(10):e70591. doi: 10.1002/bmc.70591 (PMC13474908; doi:10.1002/bmc.70591)
Supplement: Supplementary file 1 [file BMC-40-e70591-s001.docx]

**A rapid and sensitive LC-MS/MS method for quantification of NDSRI in Melatonin Drug product via controlled In-situ NDSRI formation**

Jaya Yalamanchili^a^, Ganja Himavathi^a*^, Salakolusu Suresh^b^

*^a^Department of Chemistry, GITAM School of Science, GITAM Deemed to be University, Vishakhapatnam-530045, Andhra Pradesh, India.*

*^b^Analytical Discovery Chemistry, Aragen Life Sciences Pvt. Ltd., IDA Nacharam, Hyderabad, 500076, India*

**Corresponding author:** [**hganja@gitam.edu**](mailto:hganja@gitam.edu)

**Supporting data-1:** Chromatograms and calculations

| **Section** | **Description** | **Pg. no.** |
| --- | --- | --- |
| (A) | Precision chromatograms | 1–3 |
| (B) | Intermediate precision chromatograms | 3–5 |
| (C) | Precision and intermediate precision calculations | 5 |
| (D) | Linearity chromatograms | 6–7 |
| (E) | Linearity calculations | 8 |
| (F) | Accuracy chromatograms | 9–11 |
| (G) | Accuracy calculations | 12 |

***A: Precision Chromatograms***

***Spike sample preparation-1***

***Spike sample preparation-2***

***Spike sample preparation-3***

***Spike sample preparation-4***

***Spike sample preparation-5***

***Spike sample preparation-6***

***B: Intermediate precision Chromatograms***

***Spike sample preparation-1***

***Spike sample preparation-2***

***Spike sample preparation-3***

***Spike sample preparation-4***

***Spike sample preparation-5***

***Spike sample preparation-6***

***C: Precision and Intermediate precision Calculations***

| **Parameter** | **Sample description** | **Content (ppm)** |
| --- | --- | --- |
| **Precision** | Spike sample preparation-1 | 1.7254 |
|  | Spike sample preparation-2 | 1.7135 |
|  | Spike sample preparation-3 | 1.7222 |
|  | Spike sample preparation-4 | 1.7317 |
|  | Spike sample preparation-5 | 1.7185 |
|  | Spike sample preparation-6 | 1.6960 |
| **% RSD (n=6)** | | **0.7** |
| Intermediate precision | Spike sample preparation-1 | 1.7373 |
|  | Spike sample preparation-2 | 1.7597 |
|  | Spike sample preparation-3 | 1.7720 |
|  | Spike sample preparation-4 | 1.8005 |
|  | Spike sample preparation-5 | 1.7191 |
|  | Spike sample preparation-6 | 1.7782 |
| **% RSD (n=6)** | | **1.6** |
| **% RSD (n=12)** | | **1.8** |

***D: Linearity Chromatograms***

***Level-1***

***Level-2***

***Level-3***

***Level-4***

***Level-5***

***E: Linearity Calculation***

| **Parameter** | **Sample description** | **Concentration (ppm)** | **Peak area** |
| --- | --- | --- | --- |
| **Linearity** | Linearity level-1 | 0.18 | 81746 |
|  | Linearity level-2 | 0.9 | 436486 |
|  | Linearity level-3 | 1.35 | 676486 |
|  | Linearity level-4 | 1.8 | 897648 |
|  | Linearity level-5 | 2.7 | 1424398 |
| **Correlation coefficient** | | **0.9983** | |

N-Nitroso melatonin

| Concentration (ppm) | Peak area |
| --- | --- |
| 0.18 | 81746 |
| 0.9 | 436486 |
| 1.35 | 676486 |
| 1.8 | 897648 |
| 2.7 | 1424398 |

***F: Accuracy Chromatograms***

***Spike sample at LOQ preparation-1***

***Spike sample at LOQ preparation-2***

***Spike sample at LOQ preparation-3***

***Spike sample at 100% specification preparation-1***

***Spike sample at 100% specification preparation-2***

***Spike sample at 100% specification preparation-3***

***Spike sample at 150% specification preparation-1***

***Spike sample at 150% specification preparation-2***

***Spike sample at 150% specification preparation-3***

***G: Accuracy: Calculations***

| **Parameter** | | **Sample description** | **Content (ppm)** | **% Recovery** |
| --- | --- | --- | --- | --- |
| **Accuracy** | | Spike sample at LOQ preparation-1 | 0.1629 | **90.52** |
|  |  | Spike sample at LOQ preparation-2 | 0.1659 | **92.18** |
|  |  | Spike sample at LOQ preparation-3 | 0.1608 | **89.32** |
| **% RSD (n=3)** | | | | **1.6** |
| **Accuracy** | | Spike sample at 100% preparation-1 | 1.7373 | **95.86** |
|  |  | Spike sample at 100% preparation-2 | 1.7597 | **95.20** |
|  |  | Spike sample at 100% preparation-3 | 1.7720 | **95.68** |
| **% RSD (n=3)** | | | | **0.4** |
| **Accuracy** | Spike sample at 150% preparation-1 | | 2.3523 | **87.12** |
|  | Spike sample at 150% preparation-2 | | 2.3519 | **87.11** |
|  | Spike sample at 150% preparation-3 | | 2.3030 | **85.30** |
| **% RSD (n=3)** | | | | **1.2** |
